# Quantitative analysis of F-actin alterations in adherent human mesenchymal stem cells: Influence of slow-freezing and vitrification-based cryopreservation

**DOI:** 10.1371/journal.pone.0211382

**Published:** 2019-01-25

**Authors:** Yannik Müllers, Ina Meiser, Frank Stracke, Iris Riemann, Franziska Lautenschläger, Julia C. Neubauer, Heiko Zimmermann

**Affiliations:** 1 Department of Cryo- and Stem Cell Technology, Fraunhofer Institute for Biomedical Engineering (IBMT), Joseph-von-Fraunhofer-Weg 1, Sulzbach, Germany; 2 Division of Cytoskeletal Fibers, Leibniz Institute for New Materials, Campus D2 2, Saarbrücken, Germany; 3 Chair for Experimental Physics, Saarland University, Saarbrücken, Germany; 4 Fraunhofer Project Centre for Stem Cell Process Engineering, Neunerplatz 2, Würzburg, Germany; 5 Chair for Molecular and Cellular Biotechnology, Saarland University, Saarbruecken, Germany; 6 Faculty of Marine Science, Universidad Católica del Norte, Coquimbo, Chile; Peking University Third Hospital, CHINA

## Abstract

Cryopreservation is an essential tool to meet the increasing demand for stem cells in medical applications. To ensure maintenance of cell function upon thawing, the preservation of the actin cytoskeleton is crucial, but so far there is little quantitative data on the influence of cryopreservation on cytoskeletal structures. For this reason, our study aims to quantitatively describe cryopreservation induced alterations to F-actin in adherent human mesenchymal stem cells, as a basic model for biomedical applications. Here we have characterised the actin cytoskeleton on single-cell level by calculating the circular standard deviation of filament orientation, F-actin content, and average filament length. Cryo-induced alterations of these parameters in identical cells pre and post cryopreservation provide the basis of our investigation. Differences between the impact of slow-freezing and vitrification are qualitatively analyzed and highlighted. Our analysis is supported by live cryo imaging of the actin cytoskeleton via two photon microscopy. We found similar actin alterations in slow-frozen and vitrified cells including buckling of actin filaments, reduction of F-actin content and filament shortening. These alterations indicate limited functionality of the respective cells. However, there are substantial differences in the frequency and time dependence of F-actin disruptions among the applied cryopreservation strategies; immediately after thawing, cytoskeletal structures show least disruption after slow freezing at a rate of 1°C/min. As post-thaw recovery progresses, the ratio of cells with actin disruptions increases, particularly in slow frozen cells. After 120 min of recovery the proportion of cells with an intact actin cytoskeleton is higher in vitrified than in slow frozen cells. Freezing at 10°C/min is associated with a high ratio of impaired cells throughout the post-thawing culture.

## Introduction

The application of human stem cells is a promising approach for various fields in regenerative medicine. In particular, patient’s autologous mesenchymal stem cells (hMSCs) have the potential to overcome limitations of conventional transplantations, such as transplant shortage or immune rejections [1]. Successful treatment of osteoarthritis [2], cartilage defects [3] and cardiac disease [4] have been reported so far, where a constant supply of stem cells is an unavoidable prerequisite for those medical approaches. Up until now, cryopreservation is the only option for storing viable cells in a stable manner for long periods of time and enable generation of stocks for future use.

In general, there are two basic techniques for cryopreservation; slow rate freezing and vitrification. During slow rate freezing, crystallization of the extracellular medium occurs, while the water inside the cell is still liquid [5]. Consequently, osmotic pressure rises in the extracellular medium due to increased concentration of solutes. Depending on the cooling rate, two different damaging mechanisms arise; cells either lose too much water, which leads to harming solution effects, or intracellular ice formation occurs [6] which in turn leads to a harmful loss of liquid intracellular water too. To counteract this, freezing medium includes permeable cryoprotective agents, such as dimethyl sulfoxide (DMSO), that reduce the amount of ice formation within cells [7]. In contrast, when using vitrification, no ice is formed at all leading to a completely glassy sample state. Hence, neither osmotic imbalances due to extracellular crystallization nor cell injuries from intracellular ice formation occur. To successfully vitrify cells, the glass transition temperature must be passed before crystallization starts. This can be achieved by using highly viscous media to increase the glass transition temperature and ultra-fast cooling rates [8]. Due to limitations of the applicable heating rate, devitrification and recrystallization with its harming effects can occur during the rewarming process of vitrified and slow-frozen samples. The decision of which cryopreservation method is superior strongly depends on characteristics of the sample. For most suspended cells, slow-freezing delivers consistent results and is easy to perform. However, in some cases vitrification shows better results than slow freezing in regard to post-thaw survival rate, morphology and successful implantation of cryopreserved human embryos [9]. Cryopreservation of adherent cell systems remains a challenge in current research [10–13] but there is evidence that vitrification is generally more successful [14–16].

Besides cell viability, the success of cryopreservation can also be assessed in terms of the functionality of cells after thawing. The functionality of cryopreserved hMSCs as a model for medical applications is of special interest and has been investigated in several studies. One study focused on the immunosuppressive effect of hMSCs whereby limited ability to suppress T cell proliferation was observed post thawing [17]. Furthermore, freeze-thawed hMSCs in contrast to fresh hMSCs were lysed by T cells [18]. Apart from that, it was found that cryopreserved MSCs can reveal inhibited homing activity in the context of inflammation treatment [19]. Depending on pre-freeze senescence, slow freezing of suspended, bone marrow derived hMSCs resulted in reduced post thaw cell activity and growth rate [20].

A variety of essential cellular functions such as adhesion [21], migration [22], proliferation [23] and even differentiation [24] are controlled by the actin cytoskeleton. Thus actin cytoskeleton status is an important factor to generate ready to use functional, cryopreserved stem cells. Recent literature has reported cryopreservation induced alterations of hMSCs actin cytoskeleton including reduction of F-actin content [25], impaired membrane-cytoskeleton interaction [26] and altered elastic modulus [27]. However, data from these studies relied on cryopreservation of suspended cells. Therefore, cell detachment before cryopreservation and re-attachment of the cells for post-thaw analysis was not assessed. Both of these steps highly impact the actin cytoskeleton independently from cryopreservation, an aspect which can be avoided by directly freezing the cells in their adherent state. Furthermore, adherent cryopreservation enables comparison of the actin cytoskeleton in identical cells pre-freeze and post-thaw. Only one previous study has assessed the actin cytoskeleton of hMSCs cryopreserved in their adherent state so far whereby Xu and colleagues [28] cryopreserved adherent hMSCs by slow freezing at 1°C/min and 10°C/min, reporting qualitative alterations in actin morphology highly dependent on the rate of freezing. To further these investigations, we developed a method to quantitatively analyze the actin cytoskeleton in identical cells before adherent cryopreservation and after thawing. Our results are supported by high-resolution live-cryo microscopy during freezing. This method enables us to objectively compare actin cytoskeleton preservation between slow freezing and vitrification. Furthermore, the quantitative analysis reveals information about possible origins for actin cytoskeletal damage during cryopreservation and thus offers approaches for improvement.

## Materials and methods

### Human wharton’s jelly MSC culture

Wharton`s jelly derived hMSCs (PromoCell GmbH, Heidelberg, Germany) were cultured and expanded in culture medium: DMEM/F12, containing 10% FCS, 1% penicillin/streptomycin and 0.05% basic fibroblast growth factor (all from Gibco, Karlsruhe, Germany). Culture medium was exchanged every two or three days. At 80% confluency, cells were passaged, using 2.5 ml trypsin (Gibco, Karlsruhe, Germany) for 3 min and seeded for experiments as described below.

### Slow rate-freezing

For cryopreservation, hMSCs were seeded on culture-dishes (μ-Dish 35 mm, high Grid-500, ibidi, Matrinsried, Germany). Before freezing, culture medium was replaced by 500 μl Cryostor CS-10 (containing 10% DMSO, BioLife Solutions, Bothell, WA, USA). Cells were placed into a 4°C-precooled, programmable freezer (Ice-Cube 14S, SY-LAB, Neupurkersdorf, Austria) and frozen through temperature reduction at a cooling rate of 1°C/min or 10°C/min respectively. At -80°C, samples were exposed to liquid nitrogen (LN_2_) in order to mimic storage below the glass transition temperature of water for a few seconds. Upon thawing, 2ml of culture medium pre-warmed to 37°C was pipetted onto the cells. After washing with culture medium to remove DMSO, cells were incubated at 37°C, with 5% CO_2_ for recovery.

### Vitrification

For vitrification, “TWIST”-substrates [15] were used to prevent cell line contamination due to direct exposure to LN_2_. The “TWIST”-substrate consists of two culture dishes (μ-Dish 35 mm, high, ibidi, Matrinsried, Germany), one containing the adherent cell culture, and another one with its bottom removed. Bottom sides of both dishes are agglutinated resulting in two compartments separated by a thin plastic layer. This technique allows rapid cooling while avoiding direct contact between cells and LN_2_. Before vitrification as well as for warming, the osmolarity of the culture was adjusted stepwise via two vitrification solutions (V1, V2) and two warming solutions (W1, W2) according to Table 1. DMSO, ethylene glycol (EG) and sucrose where provided from Sigma-Aldrich, Schnelldorf, Germany. Culture medium in the cell compartment of the TWIST substrate was replaced by 500 μl V1. After 1 min incubation at room temperature (RT), V1 was replaced by 500 μl V2. Following 5 s incubation, V2 was aspirated, the substrate twisted and LN_2_ filled into the respective compartment. Right after LN_2_ was vaporized, the substrate was twisted again and 3 ml of 37°C pre-warmed W1 was pipetted onto the cells for warming. After 1 min incubation, W1 was replaced by 1 ml W2 for additional 5 min incubation. W2 was replaced by culture medium and cells were incubated for recovery.

**Table 1:** Composition of vitrification and warming solutions.

Solution	DMSO	EG	Sucrose (1 molar^a^)	Culture medium
**V1**	10%	10%	-	80%
**V2**	20%	20%	30%	30%
**W1**	-	-	20%	80%
**W2**	-	-	10%	90%

^a^diluted in culture medium

### Fluorescence staining of F-actin in living cells

Either SIR-actin (Spirochrome, Stein am Rhein, Switzerland) or the Actin-GFP BacMam 2.0 system (Life Technologies Corp, Eugene, OR, USA) were used for F-actin live-staining. Cells were incubated for 16 h with 50 nM SIR-actin, or 1% Actin-GFP respectively, in culture medium at 37°C, with 5% CO_2_.

### Fluorescence staining of phosphatidylserine

To determine apoptosis, phosphatidylserine was detected by 20 min incubation at RT with 1% Annexin-V Alexa Fluor 488 conjugate (Life Technologies Corp, Eugene, OR, USA) in culture medium and subsequent rinsing with pure culture medium.

### Confocal microscopy for quantitative F-actin comparison before and after cryopreservation

10,000 hMSCs/ml were seeded on μ-dishes and incubated for 5 h to ensure adhesion. F-actin was stained with SIR-actin, phosphatidylserine with Annexin-V Alexa Fluor 488 conjugate. Fluorescence signals of 15 single cells were measured per experiment with a confocal laser scanning microscope (CLSM) model TCS SP8 (Leica Microsystems, Wetzlar, Germany). The fluorescence was measured at excitation wavelengths 652 nm and 488 nm and emission wavelength 647 nm and 525 nm for SIR and AF-488 respectively. For imaging, a 63X/1.40 oil-immersion objective was used. To image the full depth of a cell, Z-stacks were collected at 1μm intervals. Those Z-stacks were projected on a 2D-image via maximum intensity projection for analysis. For relocation of identical cells after cryopreservation, the position of each cell was recorded. Then, cells were cryopreserved by either slow-freezing or vitrification. After thawing, cells were incubated for 0 min, 15 min or 120 min recovery and subsequently fixed using Cytofix solution (BD Biosciences, Heidelberg, Germany). Phosphatidylserine staining was repeated and the fluorescent signals of the identical 15 cells were detected again using the same microscope settings detailed above. As a control, the experimental procedure was also performed with untreated cells (no cryopreservation). Experiments were repeated three times (N = 3, n = 45).

### Two photon microscopy for qualitative F-actin imaging during complete freeze-thaw cycle

5,000 hMSCs/ml were seeded onto coverslips in 6-well plates, (Greiner Bio-One GmbH, Frickenhausen, Germany) containing culture medium. After an incubation of 5 h, F-actin was stained with the Actin-GFP BacMam 2.0 system. For cooling and thawing, a cryo stage MDS 600 (Linkam Scientific Instruments, Tadworth, UK) was used. A coverslip was fixed onto the cryo stage with a droplet of water. Onto this coverslip, the coverslip with adherent hMSCs was fixed cell-side down, using 5 μl Cryostor CS-10. The cryo stage was optically linked to a two photon microscope, an inverted scanning microscope (LSM 510 META NLO, Carl Zeiss, Jena, Germany) was operated with a Coherent Chameleon Ultra mode-locked laser (Coherent, Santa Clara, CA, USA). For details of this imaging method see Doerr and coworkers’ report [29]. Two photon excitation of actin-GFP was performed at 900 nm. For detection, a 500 nm—550 nm band-pass filter was applied. A 40X/1.30 oil-immersion objective was used for imaging. After a reference imaging of a cell`s actin cytoskeleton at 23°C, freezing was applied with reduction in temperature at either 1°C/min or 10°C/min. Images of the actin cytoskeleton were captured in 5°C steps. After reaching -80°C, cells were rapidly thawed with 100°C/min up to 37°C. Immediately, 5 min, 10 min and 15 min after thawing, cells were imaged again. Three individual cells were examined per cryopreservation approach, the obtained images were evaluated qualitatively.

### Analysis of apoptosis

The intensity of the Annexin-V Alexa Fluor 488 conjugate fluorescence was measured for every single cell, using ImageJ (NIH, Bethesda, MD, USA). To distinguish between apoptotic negative and apoptotic positive cells, the standard deviation of the fluorescent intensity was analyzed. Cells with standard deviation higher than 6000 arbitrary units (a.u.) were defined as apoptotic positive (S1 Fig). Microscope settings were standardized for apoptosis measurements and images were not processed afterwards. We did not detect any apoptosis signal in control measurements before cryopreservation.

### Quantification of the actin cytoskeleton

For image analysis of the actin cytoskeleton, the software Filament Sensor [30] was used. Settings except of *minimum mean value* (set to 5) were kept default. The software measures length, width, orientation (angle to the horizontal) and position of every single filament of a cell`s actin network. Based on these measured variables, we defined three characteristic parameters to describe the actin cytoskeleton as a whole. These characteristic parameters are the circular standard deviation ν, the F-actin-content F and the average filament-length L. F and L are calculated as follows:

$$F\;=\;\sum_{i\;=\;1}^{n}l_{i}w_{i}\;and\;L\;=\;\frac{1}{n}\sum_{i}l_{i}$$

with “n” as the total number of measured filaments, “l_i_” the length of filament “i” and “w_i_” the width of filament “i”. The circular standard deviation ν is a measurement of the isotropy of filament`s orientation, which has for instance been used to characterize keratin networks [31]. For circular statistics, the angles α_1_, α_2_,…., α_n_ of the “n” measured filaments are interpreted as unit vectors with the respective orientation. These vectors can be added up to one resulting vector. The length “R”, normalized by “n”, of this resulting vector is then a measurement for the isotropy of filament orientation in the actin cytoskeleton. Based on these considerations, “R” can be written as:

$$nR\;=\;\sqrt{{\left(\sum_{i\;=\;1}^{n}\text{sin}\;\alpha_{i}\right)}^{2}+{\left(\sum_{i\;=\;1}^{n}\text{cos}\;\alpha_{i}\right)}^{2}}$$

By convention, the circular standard deviation “ν” is calculated from “R”:

$$\nu \;=\;\sqrt{-2ln\;(R)}$$

Small values of “ν” indicate a high degree of parallelism of actin filaments, high values a branched network. In S2 Fig. different, representative F-actin morphologies are related to the corresponding values of the introduced parameters.

### Classification based on actin cytoskeletal alterations

The characterizing parameters described above are calculated in identical cells before and after cryopreservation. Then, the percentage alteration Δ of each parameter is determined for every cell as

$$\Delta_{F}\;=\;100(\frac{F_{a}}{F_{b}}-1)$$

The indexes imply the value of the respective parameter after (a) and before (b) cryopreservation. Alterations of the remaining parameters Δ_ν_ and Δ_L_ are determined. These alterations are summarized as a total alteration Δ_T_ of the actin cytoskeleton via

$$\Delta_{T}\;=\;\frac{1}{3}(|\Delta_{F}|+|\Delta_{L}|+|\Delta_{\nu }|)$$

This parameter Δ_T_ -combined with the apoptosis measurement as an indicator for cryo-influence-, was employed to classify each cell in either class I (no actin disruption), class II (light actin disruption) or class III (severe actin disruptions). Apoptosis-negative cells are classified in class I. Apoptosis-positive cells are classified depending on Δ_T_ in class I (Δ_T_ < 10%), class II (10% < Δ_T_ < 20%) or class III (Δ_T_ > 20%). Cells that have lost surface contact during cryopreservation are classified as “detached cells”

### Statistical analysis

Similarly to classification, the percentage deviations of all three characterizing parameters are calculated in identical cells before and after cryopreservation. Data from the three replicates of each respective condition are taken into account for calculation of medians and subsequent statistical analysis. Medians are compared between the three applied cryopreservation routines (slow freezing with 1°C/min, 10°C/min and vitrification) and non-cryopreserved control cells for three different times post thawing (0, 15, and 120 min). Significant differences between the data sets are evaluated by Mann-Whitney U test which is more robust with regard to outliners than common t test. Equality of medians was used as null hypotheses.

## Results

### Cytoskeletal disruption during recovery after freezing at 1°C/min

**Fig 1:**
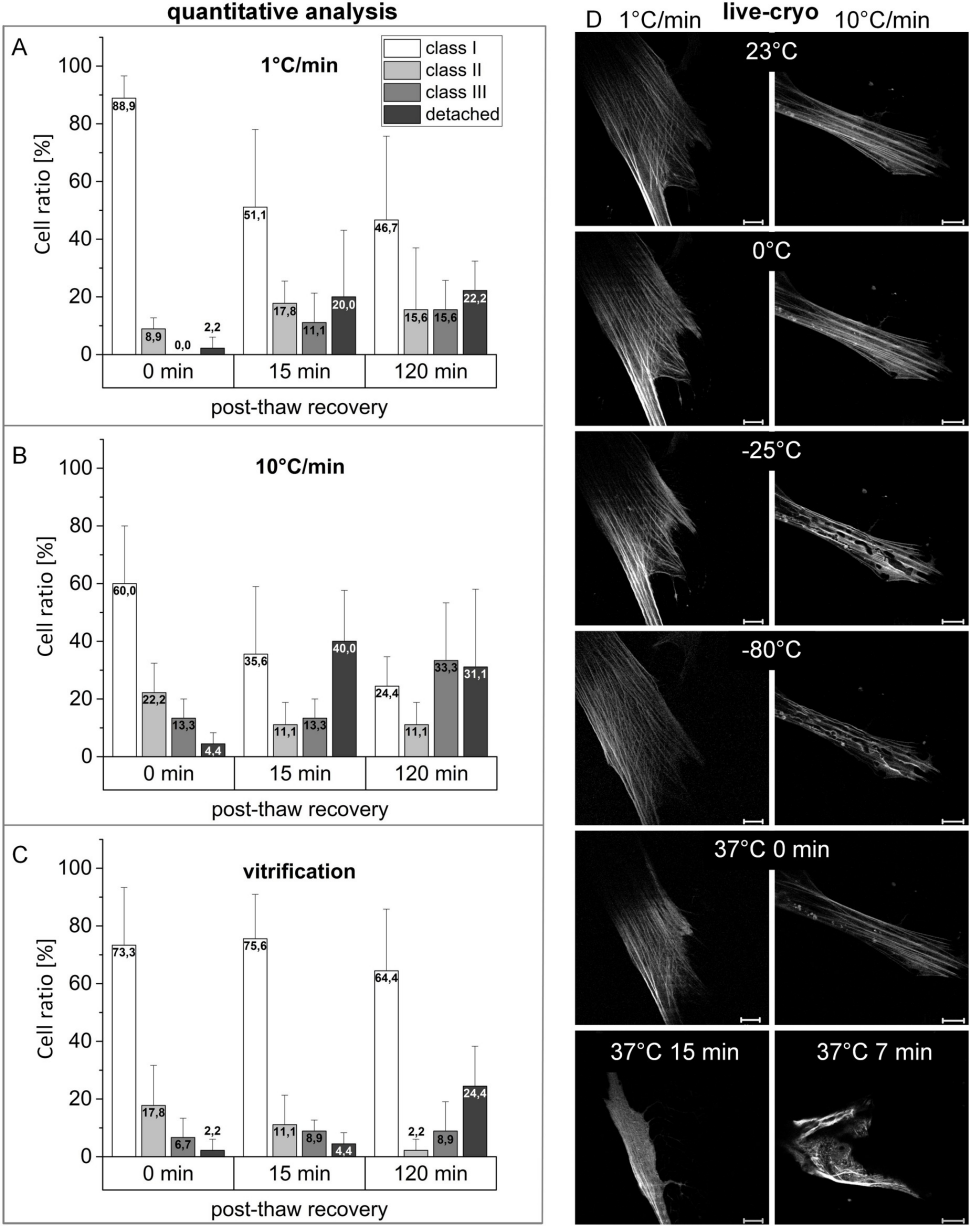
Time and frequency of actin-cytoskeleton disruption depending on cryopreservation strategy. Cryopreserved hMSCs by slow freezing at 1°C/min (A), 10°C/min (B) and vitrification (C) are classified depending on quantified cytoskeletal disruption in class I (no disruption), class II (slight disruption), class III (severe disruption), and detached cells. Bars show mean value with standard deviation from three experimental replicates (N = 3, n = 45), 0, 15, and 120 min after thawing. Representative live-cryo images from two photon microscopy (D) show the actin cytoskeleton qualitatively during slow freezing and after subsequent thawing (37°C). The presented images originate from one out of three measurement with each cooling rate. For better visualization, contrast and brightness of the presented images were adjusted. Scale bars are 20 μm.

Immediately after thawing, 88.9% of hMSCs slow-frozen at 1°C/min revealed an unchanged actin morphology without any disruptions (Fig 1A). No class III cells were detected in this condition. However, after 15 minutes recovery at 37°C, the ratio of cells in class I (whereby F-actin morphology is similar in comparison to non-frozen cells) was reduced to below 52%. 11.8% of cells even exhibited severe disruptions to F-actin morphology and 20% of the cells became detached. After 120 min recovery the ratio of cells in class I and II were slightly reduced while cells in class III and detached cells slightly increased.

Live-cryo images during slow-freezing at 1°C/min, followed by thawing (Fig 1D left column), also indicated entire preservation of F-actin structure, confirming the preceding, quantitative analysis. 15 minutes after thawing, the observed cell then lost its cytoskeletal structure.

### Cytoskeletal disruption during freezing at 10°C/min

Slow-freezing at 10°C/min leads to actin cytoskeletal disruptions in 35.5% of hMSCs immediately after thawing (Fig 1B) with 4.4% of the cells becoming detached. After 15 min of recovery, the ratio of detached cells dramatically increased to 40% with only 35.6% of cells showing regular F-actin morphology. For cells fixed after 120 min of recovery, the ratio classified in class I is below 25%, 33.3% reveal severe actin disruptions while 31.1% of the cells were detached.

The right column in Fig 1D shows a cell during slow-freezing with 10°C/min. At -25°C, intracellular ice formation was observed causing distortion of actin filaments which is clearly observed at -80°C. After thawing, the observed damages are first reduced, but increase again with progressing post-thaw incubation. Two additional cells were imaged during 10°C/min freezing which did not display intracellular ice formation.

### Partial recovery of actin cytoskeleton in vitrified hMSCs within 15 min post thawing

Vitrifying hMSCs preserves actin cytoskeletal structure in 73.3% of the cells measured immediately after thawing (time point 0 min, Fig 1C). In contrast to slow-frozen cells, this ratio increases during recovery to 75.6% after 15 min and the ratio of detached cells and those classified as class III was slightly increased. However, 120 min after thawing, 24.4% of cells were detached while the ratio of cells with actin disruptions (class II and III) was reduced.

### Increased isotropy of actin fibers after cryopreservation

**Fig 2:**
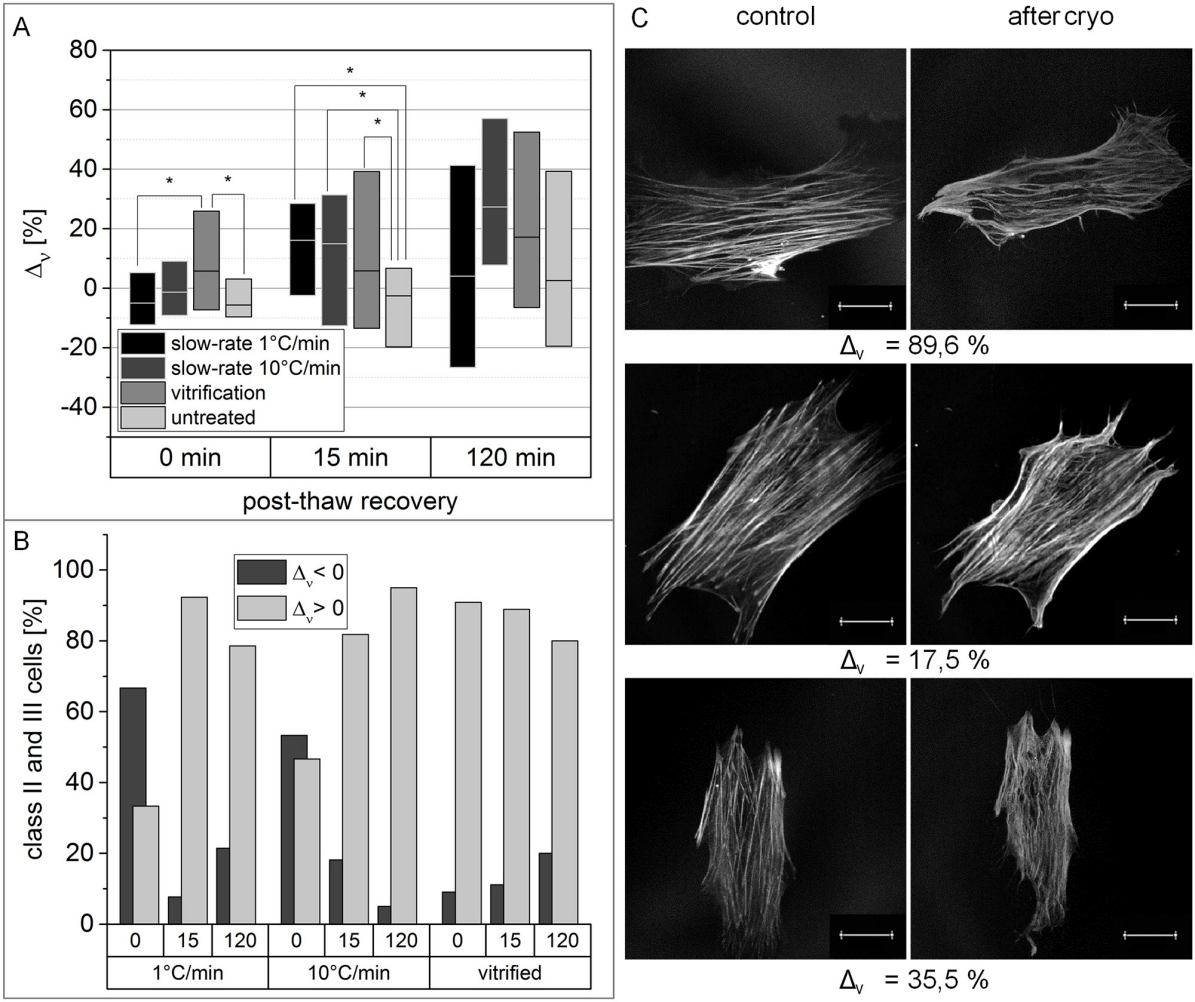
Circular standard deviation is significantly increased in the actin cytoskeleton of cryopreserved hMSCs. (A) Boxplots show the difference of circular standard deviation between identical cells before and after cryopreservation with varying recovery time. Non-cryopreserved cells have been incubated for the respective recovery time in the corresponding cryo medium as a control. Horizontal bars represent the median, box size is defined by the 25^th^ and 75^th^ percentiles. Significant differences based on Mann-Whitney-U test with p-values smaller than 0.05 are indicated by *. (B) Cells, being classified in class II or III, are separated, depending on whether circular standard deviation increased or decreased after cryopreservation. The top x-axis states the recovery time in min. (C) Representative F-actin-morphologies of hMSCs with increased circular standard deviation after cryopreservation are shown with their related Δν-value. Images were captured with CLSM. Scale bar indicates 20 μm. For better visualization, contrast and brightness of the presented images were adjusted.

Following quantification of the ratio of cells with damage to the actin cytoskeleton, the specific nature of cytoskeletal disruption was quantified. Therefore, the alterations of the three single characterizing parameters (circular standard deviation ν, filament-content F and filament-length L) were analysed separately in identical cells before and after cryopreservation. For comparison, the alterations of non-cryopreserved cells, incubated in the corresponding cryo medium for the respective recovery times, are calculated as well. Immediately after thawing, the circular standard deviation of vitrified hMSCs was significantly increased, compared to non-cryopreserved cells (Fig 2A). After 15 min of recovery, cells cryopreserved across all three methods showed a significant increase of circular standard deviation, compared with untreated cells. Vitrified and 10°C/min slow-frozen cells still showed a higher increase of circular standard deviation than non-cryopreserved cells after 120 min recovery. However, those differences are not significant based on Mann-Whitney-U test. Fig 2B emphasizes that the above described findings can mainly be attributed to cells whose actin cytoskeleton has been classified as disrupted. With the exception of slow-frozen cells assessed immediately after thawing, class II and III cells from all conditions largely showed an increase of circular standard deviation after cryopreservation. The actin cytoskeletal morphology of three hMSCs which exhibited increased circular standard deviation after cryopreservation is presented in Fig 2C whereby single actin filaments have clearly lost their straight structure and show buckling after cryopreservation.

### F-actin reduction during hMSCs cryopreservation

**Fig 3:**
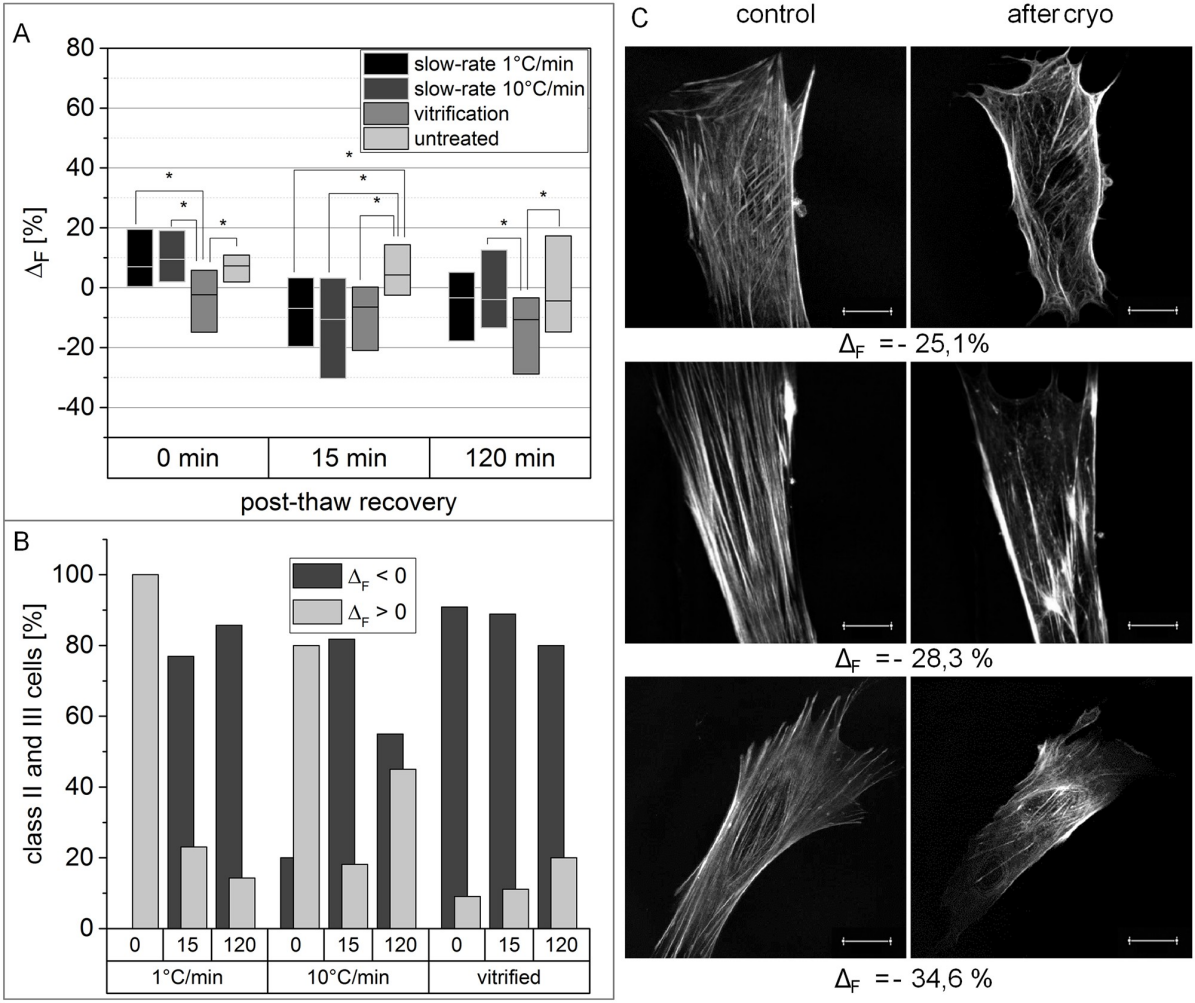
Vitrification and slow freezing result in reduced F-actin-content. (A) Boxplots show the difference of F-actin-content between identical cells before and after cryopreservation with varying recovery time. Non-cryopreserved cells have been incubated for the respective recovery time as a control. Horizontal bars represent the median, box size is defined by the 25^th^ and 75^th^ percentiles. Significant differences based on Mann-Whitney-U test with p-values smaller than 0.05 are indicated by *. (B) Cells, being classified in class II or III, are separated, depending on whether F-actin-content increased or decreased after cryopreservation. The top x-axis states the recovery time in min. (C) Representative actin cytoskeletons of hMSCs with reduced F-actin-content after cryopreservation are shown with their related Δν-value. Images were captured with CLSM. Scale bar indicates 20 μm. For better visualization, contrast and brightness of the presented images were adjusted.

The measured F-actin-content “F” was significantly reduced in vitrified hMSCs immediately after thawing (Fig 3A) in comparison to non-cryopreserved cells. After 15 min recovery, vitrified and slow-frozen cells both showed a significantly reduced F-actin-content, compared with untreated cells. 120 min recovery led to a lower F-actin-content in cells across all conditions. However, only the reduction of “F”, measured for vitrified hMSCs, is significantly higher than in non-cryopreserved cells based on Mann-Whitney-U test. Cryopreserved hMSCs with disrupted actin-cytoskeleton (class II and III) predominantly revealed negative values for Δ_F_, with the exception of freshly thawed slow-frozen cells (Fig 3B). Fig 3C shows exemplarily the actin cytoskeleton of three cells with negative Δ_F_ values. Besides a lower amount of filaments in general, the cells also exhibit holes within the meshwork after cryopreservation, leading to the measured reduction of F-actin content.

### F-actin shortening after cryopreservation of hMSCs

**Fig 4:**
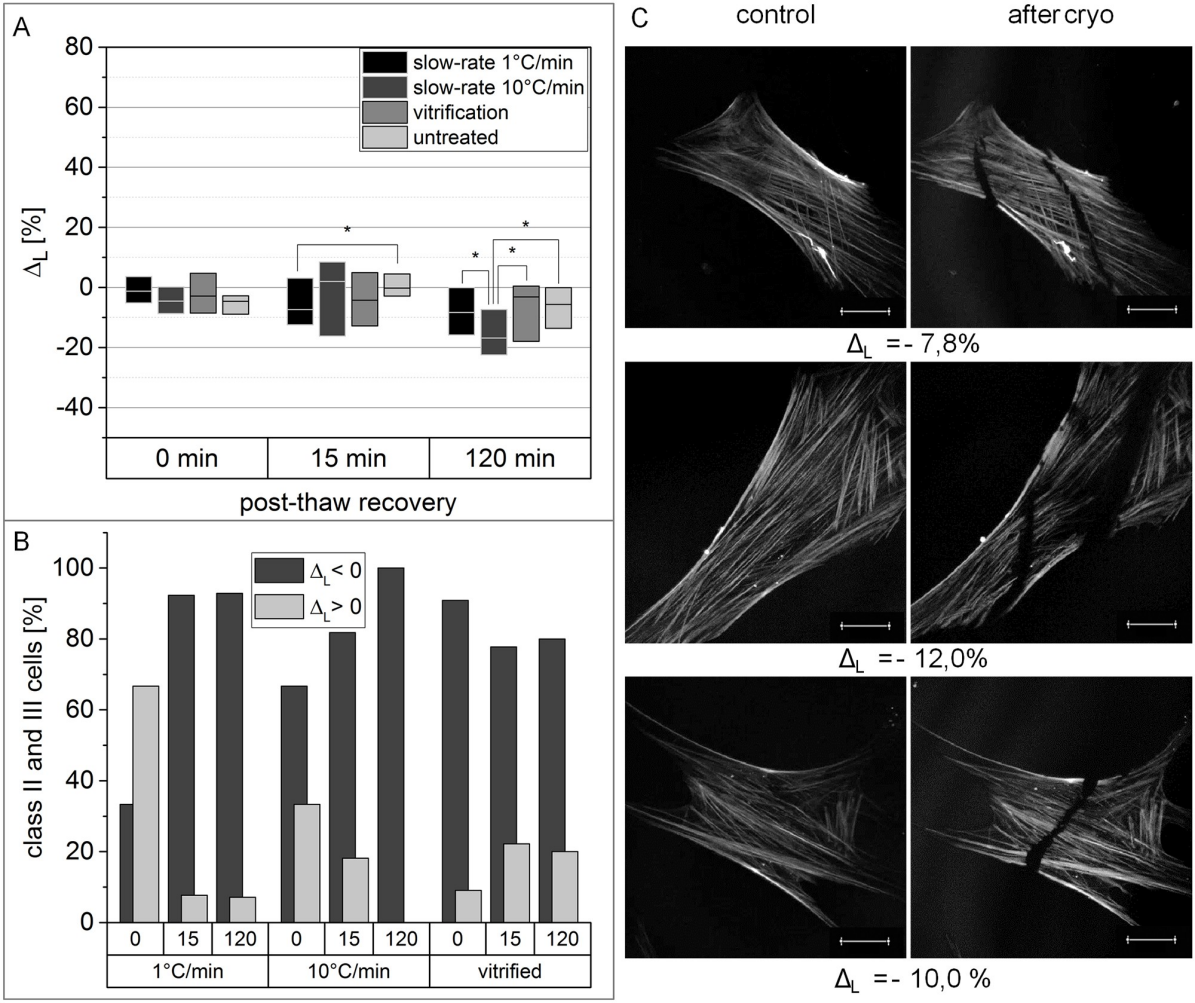
Cryopreserved hMSCs exhibit a reduced F-actin-length. (A) Boxplots show the difference of F-actin-length between identical cells before and after cryopreservation with varying recovery time. Non-cryopreserved cells have been incubated for the respective recovery time as a control. Horizontal bars represent the median, box size is defined by the 25^th^ and 75^th^ percentiles. Significant differences based on Mann-Whitney-U test with p-values smaller than 0.05 are indicated by *. (B) Cells, being classified in class II or III, are separated, depending on whether F-actin-length increased or decreased after cryopreservation. The top x-axis states the recovery time in min. (C) Representative actin cytoskeletons of hMSCs with reduced F-actin-length after cryopreservation are shown with their related Δν-value. Images were captured with CLSM. Scale bar indicates 20 μm. For better visualization, contrast and brightness of the presented images were adjusted.

Alterations of the filament-length “L” in cryopreserved hMSCs immediately after thawing are not significantly different to non-cryopreserved cells (Fig 4A). However, after 15 min recovery the filament-length of vitrified and 1°C/min slow-frozen cells was reduced, with cells slow-frozen at 1°C/min showing significant reduction in comparison to non-cryopreserved cells. In contrast, only 10°C/min slow frozen cells showed a significant reduction of filament length compared to non-cryopreserved cells after 120 min recovery. Again, the observed reduction of filament length is mainly attributed to cells exhibiting a disrupted actin cytoskeleton (Fig 4B). 100% of 10°C/min slow-frozen cells which had been categorised as class II and III after 120 min recovery, revealed a reduction of actin filament length. The same phenomenon, although less obvious, can be observed for the remaining conditions with the exception of 1°C/min slow frozen cells immediately after thawing. Cracks and holes within the actin cytoskeleton, as shown in Fig 4C, led to reduction of the measured actin filament length. The exemplarily presented morphologies of the actin cytoskeleton are highlighted after cryopreservation at 10°C/min.

## Discussion

Data of recent studies revealed an impaired functionality of cryopreserved hMSCs [17–20] which limits their potential usage in regenerative medicine. These impairments might be caused by disrupted cytoskeletal structures as reported for suspended, slow frozen hMSCs [25–27]. By cryopreservation of adherent hMSCs and integration of vitrification into cryopreservation processes our study complements these existing findings. Furthermore, we introduced a quantitative analysis based on characterization of the actin cytoskeleton of single, identical cells, thereby providing unique data on F-actin disruption at molecular level that allows us to draw conclusions about potential damaging mechanisms.

### Aspects of the applied analysis

In our study, an overall measurement of the actin cytoskeleton Δ_T_ has been introduced that enables the quantification of actin cytoskeleton alterations in identical cells pre and post cryopreservation. Due to strong variation of Δ_T_ within the measuring groups, a classification system with three Δ_T_-intervals was implemented to characterize cells according to their actin cytoskeleton alterations. Aiming at a significant comparison between identical cells before and after cryopreservation, live cells were used for the experiments. Depending on the freezing method and the allotted duration of post-thaw cultivation, living cells spent different times in physiological conditions between control measurement and fixation. During this time window (up to 120 min), cells exhibited their native activity including migration, adhesion and proliferation all of which contribute to alter cytoskeleton morphology. Thus we basically detected two populations of cells showing high Δ_T_; one population with potentially harmful alterations resulting from cryopreservation (F-actin shortening, buckling and depolymerization) and a second population showing alterations resulting from native cellular processes in physiological conditions. To analyze the influence which cryopreservation has on the cytoskeleton, a distinction is necessary to allow a significant comparison between the different post-thawing culture durations and freezing procedures. Therefore, the precondition of a positive apoptosis staining, as proven consequence of cryo damage [32–34], was taken as a requirement for cells to be classified in class II and III. The achieved quantitative classification of hMSCs actin cytoskeleton alterations is in concordance with visual inspections (S3 Fig).

### Cooling rate affects preservation of the actin cytoskeleton and the extend of cell detachment

The ratio of cells with damaged actin cytoskeleton and the timepoint at which disruption is detectable is strongly dependent on the chosen cryopreservation procedure (Fig 1). Immediately after thawing, almost 90% of cells frozen at 1°C/min exhibited F-actin morphology comparable to non-frozen cells. These findings are contrary to those of Xu et al. [28], maybe due to differences of the cryopreservation protocol, such as longer cryopreservant medium incubation time or use of a different thawing procedure. However, after 2 h recovery, the ratio of cells showing actin disruptions increased from 8.9% to more than 30%. This result indicates that freezing at 1°C/min and subsequent thawing did not directly affect the actin cytoskeleton but triggered an active cell response when reaching physiological temperatures. Additionally, after 15 min recovery, 20% of the cells detached from the substrate. Since the actin cytoskeleton is essential for cell adhesion, the detachment might be a direct consequence of cytoskeletal damage. Conversely, 15 min after recovery of vitrified cells the ratio of cells with intact actin cytoskeleton was slightly increased. However, after 120 min recovery almost 25% of vitrified cells also detached from the substrate. The amount of cells with disrupted F-actin (class II and III) was in turn reduced, supporting the hypothesis that cytoskeletal disruption causes loss of cell viability. Slow-freezing with an applied cooling rate of 10°C/min resulted in a distinctly higher ratio of cells with actin cytoskeletal disruptions and increased numbers of detached cells compared to vitrification and 1°C/min slow freezing. In accordance with this, Xu et al. also demonstrated severe F-actin degradation at a cooling rate of 10°C/min compared to 1°C/min [28]. Fixation and analysis of cells 15 min post thawing showed higher detachment rate compared to fixation and analysis after 120 min of cells frozen with 10°C/min. Due to the high standard deviation of the detached ratio after 120 min we predict this to result to be related to experimental variation rather than to cells re-attaching.

When interpreting our data, it has to be taken into account that cryopreservation procedures have the potential to be further optimised due to the multiple variations of concentrations and combinations of different cryoprotective agents in addition to the adaption of different cooling rates which alter ice formation. A recent study reported higher survival rates of hMSCs after freezing at 10°C/min in solution with ethylene glycol, taurine and ectoine compared to freezing with 1°C/min in DMSO solution [35]. These findings however are not directly transferable to our system, since we cryopreserved adherent cells. But it indicates that preservation of cytoskeletal structures after slow-freezing might be further improved by adjusting cooling rate and cryo media.

### Cryo-induced changes of actin cytoskeleton characteristics

To quantify the exact type of cytoskeletal alterations observed, the single parameters Δ_ν_, Δ_F_ and Δ_L_ were analyzed separately. This analysis did not rely on the classification described above but included all cells equally, not least with the intention to validate the developed classification system. Although there were major differences in the amount of cells with actin cytoskeletal disruptions, the nature of those disruptions was fairly consistent across all applied cryopreservation procedures. These disruptions, including F-actin buckling, reduction of F-actin content and shortage of filament length, are hereafter discussed in detail:

### Buckling of F-actin in cryopreserved hMSCs

We measured a significantly increased isotropy of F-actin orientation after cryopreservation (Fig 2). This increase is most likely attributed to F-actin buckling, which could be observed after slow-rate freezing as well as after vitrification. Since the applied image analysis software only recognizes straight filaments, a buckled actin-filament is detected as several filaments with a big variety of orientation, leading to an increase of the calculated circular standard deviation. F-actin buckling occurs when mechanical stress is applied above a certain threshold on the actin network [36]. Regarding the process of cryopreservation, there are two possible causes for the occurrence of mechanical stress. On one hand, it is known that crystallization during cryopreservation can subsequently squeeze cells within freezing media [37] and thus is likely to induce F-actin buckling. On the other hand, osmotic stress, which is a direct consequence of slow-freezing [38], or devitrification, can lead to the phosphorylation of myosin light chain [39], resulting in increased acto-myosin contraction. Compressive stress due to myosin activity is also a potential source for F-actin-buckling [40]. Since we did not observe F-actin-buckling immediately after thawing in slow-frozen cells but only in vitrified cells, a mechanical origin seems not likely. However, during freezing at 10°C/min, intracellular ice formation occurred, which could also be responsible for buckling. So far, the phenomenon of F-actin-buckling has been described as a process, which is reversed a few seconds after stress reducing [36,41]. Nevertheless, our findings demonstrate F-actin-buckling up to at least 2 hours post-thawing, indicating a lack of or reduced reversibility. As a consequence of F-actin buckling, the elastic modulus of the actin cytoskeleton is decreased [36]. Besides this, buckled filaments are likely to sever [42] which initiates additional disruption of the actin cytoskeleton.

### Actin cytoskeleton-related consequences of intracellular ice formation and osmotic shock

Further, a reduction of F-actin content was measureable after both slow-freezing and vitrification of hMSCs (Fig 3), confirming findings of a previous study [26]. We observed two different phenomena resulting in a reduced F-actin content. First, there was a loss of actin filaments in general. Disappearance and reorganization of F-actin was already reported as a direct consequence of a hyperosmotic shock [43,44]. Thus, it is reasonable to assume osmotic changes during cryopreservation as origin for filament loss. Besides, holes within the actin cytoskeleton were detected which also leads to a reduced F-actin content. These holes can be explained by displacement of F-actin caused by intracellular ice formation. Intracellular ice formation could be observed by live-cryo imaging during freezing at 10°C/min. Intracellular ice formation is a long-known consequence of high cooling rates in slow-freezing [45]. Additionally, the average filament length of the F-actin fibers is reduced after cryopreservation (Fig 4). Both phenomena discussed above are most likely responsible for this result: At first, the detection of one buckled filament as several smaller filaments, leads to the measured shortening of actin-filaments in average. Moreover, the reduction of F-actin content is concomitant with the reduction of filament length. However, we could observe an additional cause, the appearance of cracks within the actin cytoskeleton. Since those cracks are almost exclusively observed in cells after freezing with 10°C/min, they are again most likely explained by intracellular ice formation.

### Influence of DMSO on the actin cytoskeleton

Due to its ability to reduce ice formation during cryopreservation [7], DMSO is a highly effective and widely used cryoprotective agent. The concentrations of DMSO used in our study was 10% and 20% in the accordant vitrification solutions and 10% for the cryo medium applied in slow freezing. It has been reported that depending on concentration and incubation time, DMSO can affect the actin cytoskeleton. Within 4 days of continuous DMSO exposure, cytoskeletal reorganization including development of thick, regular actin stress fibers was observed in mouse melanoma cells [46]. Incubation of mouse oocytes with 1.0M and 1.5M DMSO for 30 min resulted in irregularities and disruptions of cortical actin [47], whereas this effect was significantly reduced after an incubation in a chilled environment (at 4°C). However, most significant for our study is the finding that agrees with our control experiments, that the exposure of hMSCs to 10% DMSO for 15 min at 4°C did not cause changes of morphology and organization of the actin cytoskeleton [28]. This finding indicates that DMSO exposure is not responsible for F-actin disruptions we observed.

### Evaluation of the classification method

In a further analysis, we were able to demonstrate that the described alterations, increase of circular standard deviation following F-actin buckling, as well as reduction of F-actin content and filament length, are predominantly found in hMSCs of class II and III. The representatively shown morphologies of the disrupted actin cytoskeletons were not observed in class I cells, indicating a well-functioning separation in cells with and without actin disruptions by our classification-system.

### Potential consequences of the observed and quantified actin cytoskeleton alterations

The analyzed nature of actin cytoskeletal disruptions suggests a limited functionality of the affected cells (class II and III) after thawing. Work of other groups demonstrated that a chemically induced disruption of the actin cytoskeleton had inhibiting effects on mechanical processes like adhesion [48] and migration [7,49]. Moreover, it has already been shown that differentiation processes in hMSCs are influenced by actin cytoskeleton-modulation [50,51]. In the context of using cryopreserved hMSCs for tissue engineering in medical applications, the observed F-actin buckling is particularly problematic as due to the changes of the cytoskeleton elasticity, the interaction with the cell`s mechanical environment might be impaired. This interaction is a crucial factor to control stem cell behavior in tissue engineering [52–54]. A recent study compared the influence of slow-freezing and vitrification on hMSCs adhering to hydrogels [14]. In comparison to vitrified cells, slow frozen cells revealed significantly reduced proliferation and differentiation ability in this study. Our findings of a major increase in actin cytoskeletal disruptions in slow-frozen compared to vitrified cells, suggest that observed limitations in functionality can be considered as a direct consequence of actin disruptions. A serious limitation for application of adherently cryopreserved stem cells is cell loss. We found a maximal detachment rate of 40% of cells that were slow frozen with 10°C/min 15 min after thawing. Since the actin cytoskeleton is physically linked to the adhesion contacts of a cell [55], it is highly likely that the F-actin disruptions we report are responsible for cell loss.

### Relation between apoptosis and F-actin disruptions

Our study reveals that actin cytoskeletal disruptions in cells are generally associated with apoptosis signals. There are studies reporting both apoptosis induced by F-actin disruptions [56–59] and irregular F-actin morphology in late apoptosis signaling, including peripheral arrangement of actin structures and blebbing [60]. Since peripheral arrangement and blebbing were not observed in our study we conclude that cryopreservation-induced F-actin disruptions trigger apoptosis rather than the other way around. However, we cannot exclude that both processes are independently triggered by cryopreservation.

## Conclusions

In this study, we introduce a quantitative method to analyze F-actin alterations induced by cryopreservation of adherent cells and revealed cryo-induced alteration in the cytoskeleton of hMSCs. We found buckling of F-actin in thawed cells, probably due to squeezing of the cells during the freezing process (mechanical pressure resulting from ice crystals) or osmotic shock (cell shrinkage due to osmotic water withdrawal). Particularly after freezing with 10°C/min, actin filaments were significantly shortened. Holes and cracks inside the actin network were detected, probably related to observed intracellular ice formation. These findings lead to the assumption of a limited functionality of freeze-thawed hMSCs for medical applications applying this freezing procedure. Furthermore, impaired actin cytoskeleton post thawing is likely to cause cell loss by detachment or induction of apoptosis. Although we observed similar disruptions in vitrified cells, the ratio of cells with intact actin cytoskeleton after recovery was higher compared to slow frozen cells. Based on our results and the importance of cytoskeletal proteins in native cellular processes, we urgently suggest to integrate quantification of the actin cytoskeleton in evaluation of cryopreservation protocols. For cryopreservation of adherent hMSCs our study shows that vitrification with the TWIST-method is superior to slow freezing regarding the maintenance of cytoskeletal structures and the reduction of cell loss.

## Supporting information

S1 Fig Classification of hMSCs in apoptotic positive or negative cells. The figure shows the fluorescence signal of Annexin V Alexa Fluor 488 conjugate of 12 different hMSCs. The upper left number refers to the standard deviation of the fluorescent intensity in a.u., measured with ImageJ. Cells with standard deviation above 6000 a.u. are classified as apoptotic positive. Scale bar indicates 20 μm.
(TIF)

S2 Fig Correlation of calculated parameters and actin morphology. The figure shows the fluorescent signal of SIR-actin of 12 different hMSCs. An increase of circular standard deviation refers to more increased actin morphology. Differences in F-actin content F (displayed number in μm^2^) or filament length L (displayed number in μm) are measurable by the corresponding parameters. Scale bar indicates 20 μm. For better visualization, contrast and brightness of the presented images were adjusted.
(TIF)

S3 Fig Representative classification of hMSCs based on cryo-induced F-actin disruption. The figure shows the fluorescent signal of SIR-actin of 6 different hMSCs before and after cryopreservation. Cells without or with regular alterations of its actin cytoskeleton are classified in class I. Cells with slight actin disruptions are classified in class II. Class III actin disruptions are obviously more severe than those of class II. Scale bar indicates 20 μm. For better visualization, contrast and brightness of the presented images were adjusted.
(TIF)
