# Supplementary figures and images for: Quantitative analysis of F-actin alterations in adherent human mesenchymal stem cells: Influence of slow-freezing and vitrification-based cryopreservation
Source: PLoS One. 2019 Jan 25;14(1):e0211382. doi: 10.1371/journal.pone.0211382 (PMC6347223; doi:10.1371/journal.pone.0211382)

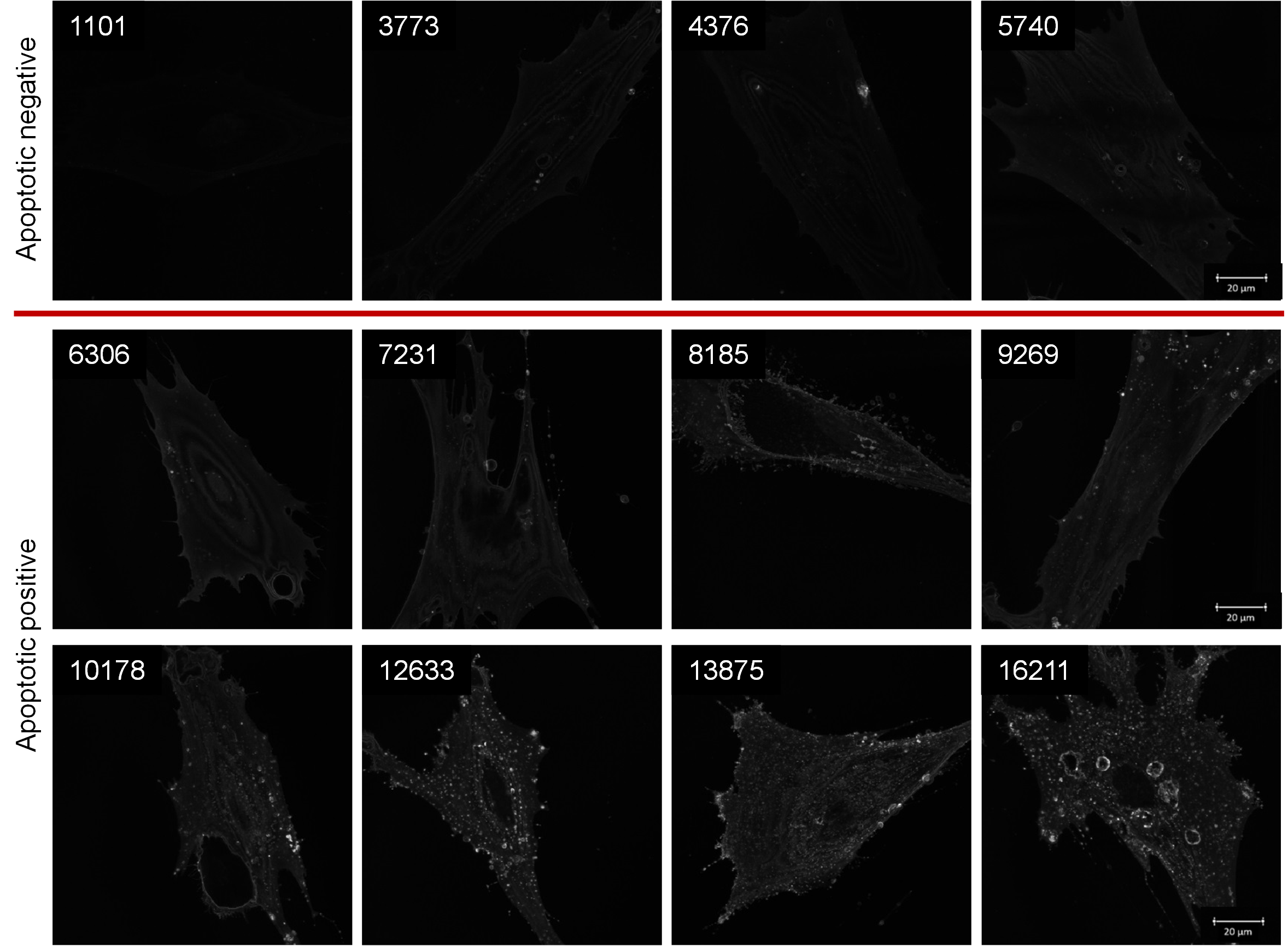

Supplement: S1 Fig — The figure shows the fluorescence signal of Annexin V Alexa Fluor 488 conjugate of 12 different hMSCs. The upper left number refers to the standard deviation of the fluorescent intensity in a.u., measured with ImageJ. Cells with standard deviation above 6000 a.u. are classified as apoptotic positive. Scale bar indicates 20 μm. (TIF) [file pone.0211382.s001.tif]

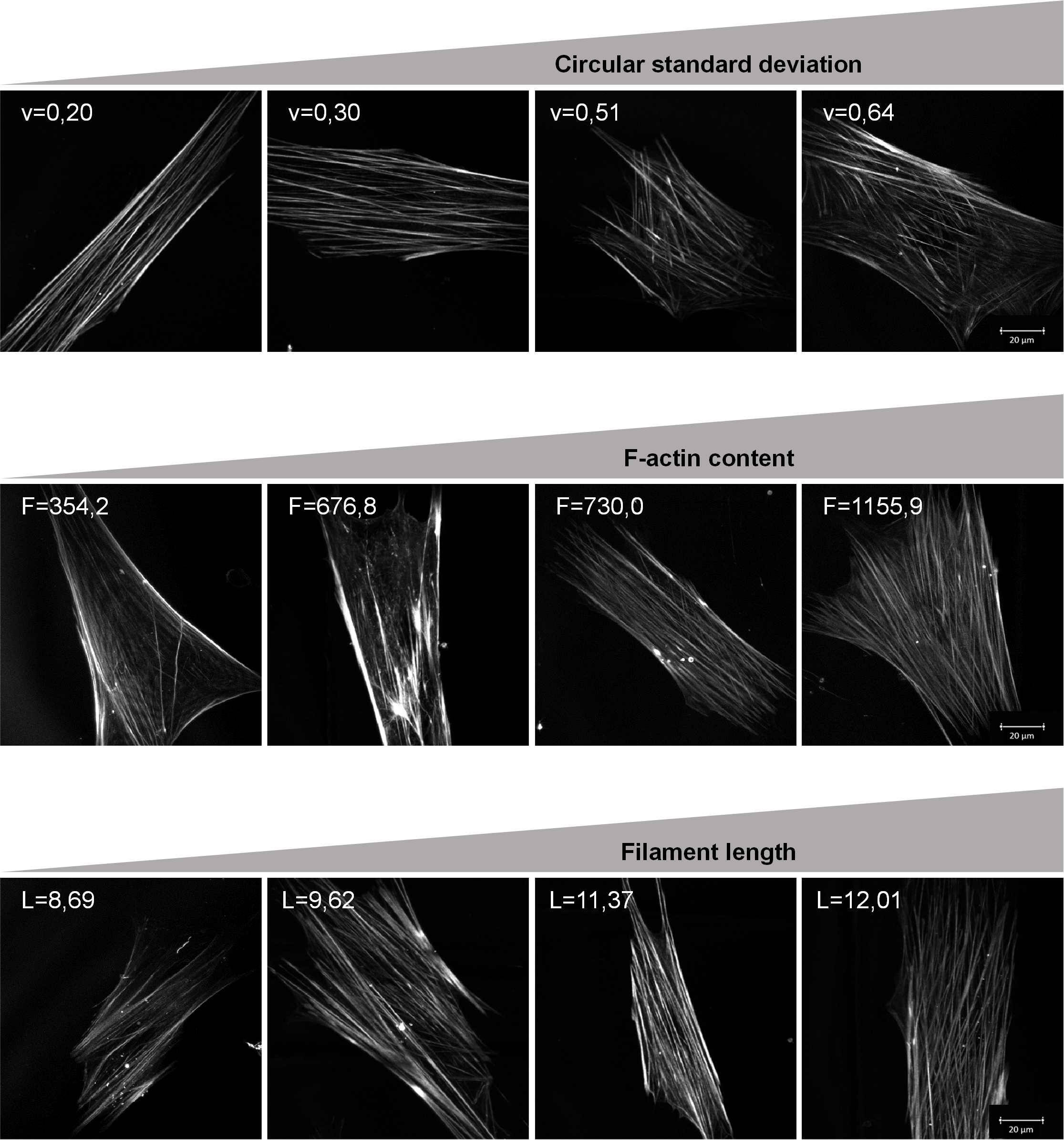

Supplement: S2 Fig — The figure shows the fluorescent signal of SIR-actin of 12 different hMSCs. An increase of circular standard deviation refers to more increased actin morphology. Differences in F-actin content F (displayed number in μm2) or filament length L (displayed number in μm) are measurable by the corresponding parameters. Scale bar indicates 20 μm. For better visualization, contrast and brightness of the presented images were adjusted. (TIF) [file pone.0211382.s002.tif]

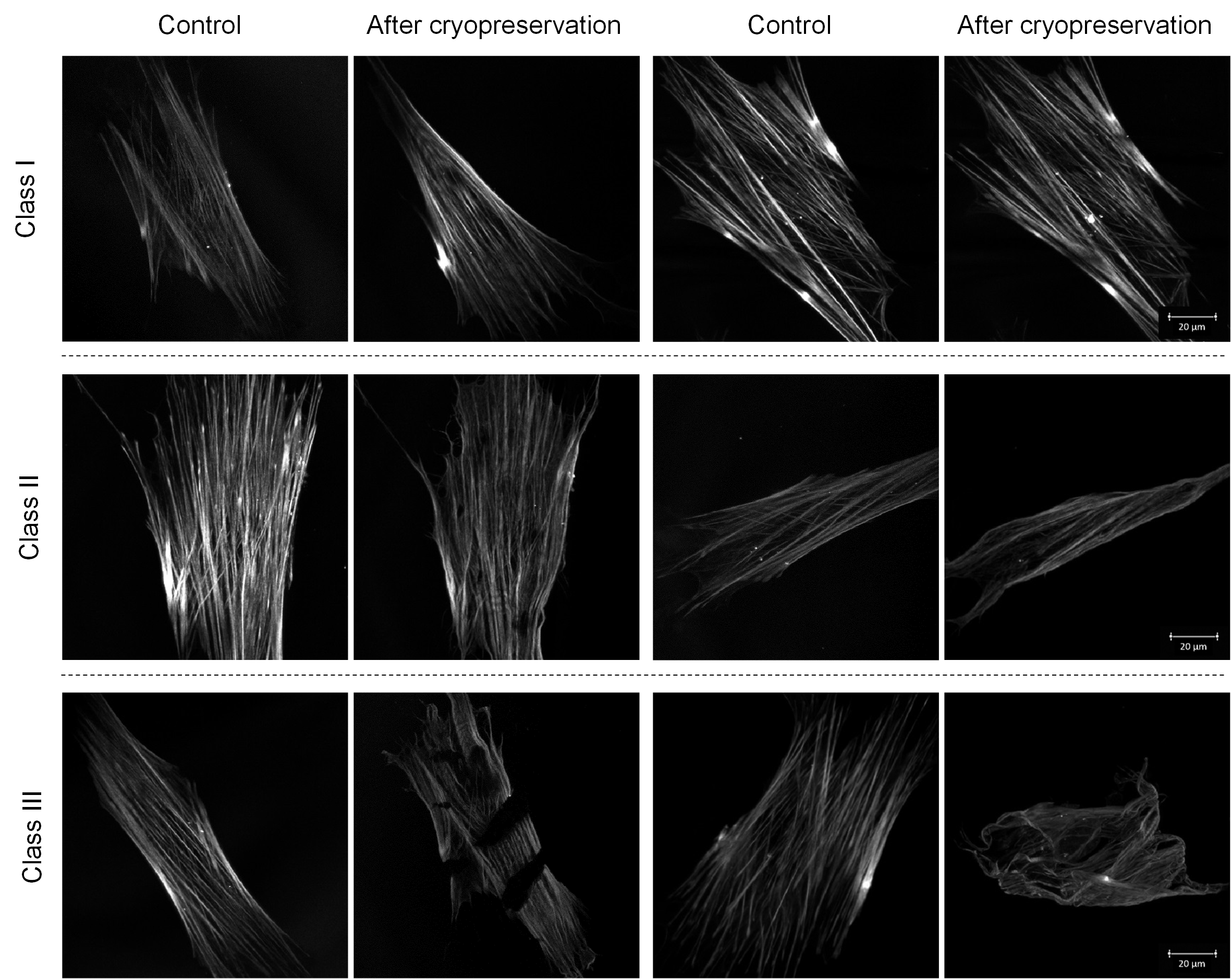

Supplement: S3 Fig — The figure shows the fluorescent signal of SIR-actin of 6 different hMSCs before and after cryopreservation. Cells without or with regular alterations of its actin cytoskeleton are classified in class I. Cells with slight actin disruptions are classified in class II. Class III actin disruptions are obviously more severe than those of class II. Scale bar indicates 20 μm. For better visualization, contrast and brightness of the presented images were adjusted. (TIF) [file pone.0211382.s003.tif]
